# Lipid Nanoparticles Loaded with Selected Iridoid Glycosides as Effective Components of Hydrogel Formulations

**DOI:** 10.3390/ma14154090

**Published:** 2021-07-22

**Authors:** Marta Dąbrowska, Izabela Nowak

**Affiliations:** Department of Applied Chemistry, Faculty of Chemistry, Adam Mickiewicz University, Uniwersytetu Poznańskiego 8, 61-614 Poznan, Poland; nowakiza@amu.edu.pl

**Keywords:** hydrogel, lipid nanoparticles, iridoid glycosides, aucubin, catalpol

## Abstract

One possibility of improving active ingredient penetration into deeper skin layers to enhance the cosmetic product effectiveness, is the application of lipid nanoparticles. The aim of the study presented in this paper was to evaluate the potential of hydrogel formulations enriched with iridoid glycosides-loaded lipid nanoparticles. Lipid nanocarriers were produced using an emulsification-ultrasonication method based on multiple emulsions. The encapsulation efficiency was determined at the level of 89% and 77% for aucubin and catalpol, respectively. The next stage was the incorporation of the obtained dispersions of lipid nanoparticles into hydrogel formulations, followed by determination of their physicochemical properties, shelf-life stability, and application properties (in vivo tests). The introduction of lipid nanoparticles increased the stabilization of the consistency of the obtained hydrogel formulations, and was confirmed by viscosity measurements. No effect of lipid nanoparticle incorporation on shelf-life stability of the hydrogels was detected. In vivo studies showed improvements in moisture content of the epidermis, transepidermal water loss, skin topography, and macrorelief parameters. In particular, a synergistic effect of the active ingredients and lipid nanoparticles on the anti-wrinkle effect, moisturizing effect, and regeneration of the protective barrier of the stratum corneum was evidenced. The attractiveness of aucubin and catalpol as cosmetic raw materials in hydrogel formulations was evidenced, especially when the iridoid glycosides were applied in the form of lipid nanoparticles.

## 1. Introduction

Intensive developments in the cosmetic product market around the world and the direction of forecasted changes in the cosmetics industry point to a continuously growing demand for cosmetics based on innovative ingredients and technologies. This is due to the increasing wealth of society and a general awareness of the composition and quality of cosmetic products used. In order to meet the growing expectations of consumers, the use of innovative raw materials and the objectively proven effectiveness of cosmetic preparations are becoming obligatory requirements. The choice of an appropriate active ingredient (AI) is of key importance, as it determines the character and effectiveness of the cosmetic activity, which also depend on the degree of release of the active substances from the matrices and the ability of the AI to permeate the barrier of the stratum corneum. One possibility of improving AI penetration into deeper skin layers and, thus, ensuring the cosmetic product effect, is the application of lipid nanoparticle supports.

Lipid nanoparticles have been used as carriers of AI in cosmetic products since 2005 [1]. Due to their structure, based on lipophilic components, lipid nanoparticles exhibit occlusive properties, reducing the level of transepidermal water loss [2,3]. Used in cosmetic formulations as carriers of active substances (in this case iridoid glycosides), they facilitate AI transport in an unchanged form to the target structures of the skin [4,5,6] and, predictably, intensify their cosmetic effects. Nanotechnology-based solutions in everyday products (also in cosmetics) raise a number of doubts about the advisability of use of new nanomaterials and their safety. Such intensive use of nanotechnology achievements brings many benefits, but it should also be realized that, at the moment, we are not able to predict what risks are posed by such intensified applications [7]. The size of nanoparticles, not only allows cosmetic active ingredients to penetrate deeper skin layers, but also raises concerns regarding: (i) the risk of penetration of the used nanomaterials into the circulation system and their accumulation in tissues and organs in the human body; (ii) the incompletely understood mechanism of action and the effects on the human body [8,9,10].

**Figure 1:**
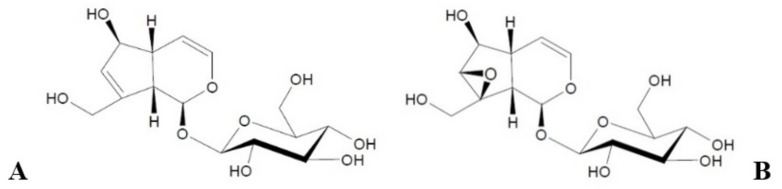
Structural formulas of aucubin (**A**) and catalpol (**B**).

Referring briefly to the cosmetic properties of the studied iridoid glycosides—aucubin and catalpol (Figure 1)—they beneficially affect the regenerative processes of the epidermis by inducing epidermal cell differentiation processes. Thus, they strengthen the protective function of the lipids of the stratum corneum [11,12]. In addition, the anti-inflammatory properties of aucubin allow the reduction of damage to the connective tissue of the skin, which occurs with age [13,14]. Aucubin also has the ability to stimulate the growth of dermal fibroblasts [11,15]. The second of the discussed iridoid glycosides—catalpol—affects processes related to the regulation of skin hydration [12,16,17]. The above characteristics illustrate the potential of the investigated iridoid glycosides for their use as cosmetic raw materials with anti-aging properties and in regenerating the barrier of the stratum corneum.

It is important to mention a key factor, namely the selection of the right cosmetic medium. It must be suitable for the introduction of lipid nanoparticles and must fully satisfy its other fundamental roles of facilitating the penetration of the active substance through the epidermal barrier, while providing primary skin care function. Hydrogels are a commonly used physicochemical form of cosmetic products. They are transparent media with a hydrophilic character, composed of up to 90% water (which is a dispersed phase), a permeation promoter, and a gelling agent, forming a gel structure. Consistency-forming components in hydrogel production are: (i) natural polymers (agar, sodium alginate); (ii) carbohydrate derivatives (hydroxyethyl cellulose—HEC, chitosan); (iii) synthetic polymers (carbomer). Hydrogels are characterized by attractive sensory textures, film-forming ability, and easy application, followed by a relatively fast absorption time [18,19]. One of the existing methods of introducing lipid nanoparticles into cosmetic products is the precise combination of lipid nanoparticles with hydrogel formulations [4,5,20], allowing the creation of a cosmetic formulation, combining the benefits of a moisturizing cosmetic base and lipid nanoparticles enriched with the selected active substances.

The literature so far on selected iridoid glycosides has been mainly focused on their pharmaceutical properties. The broadly defined cosmetic effects of aucubin and catalpol have not been thoroughly explored in the literature to date. Additionally, introducing them into lipid nanoparticles, itself a rather difficult procedure due to the hydrophilic nature of iridoid glycosides, has not been carried out by any research group. Obtaining the structures of appropriate size and charge for cosmetic applications can be treated as a step towards proving the beneficial cosmetic effect of aucubin and catalpol use as lipid carriers, supported, not only by studies of their release efficiency from hydrogels, but also by objective in vivo studies.

The aim of the study presented in this paper was to evaluate the potential of hydrogel formulations enriched with iridoid glycosides-loaded lipid nanoparticles.

## 2. Materials and Methods

### 2.1. Materials

#### 2.1.1. Reagents

The lipid nanoparticles were obtained using Softisan^®^ 100 (CAS: 91744-42-2; hydrogenated triacylglycerols as solid lipids) purchased from Sasol Germany GmbH (Hamburg, Germany), glycerol (CAS: 56-81-5), CTAB (CAS: 57-09-0; hexadecyltrimethylammonium bromide as a cationic surfactant) and Tween^®^ 80 (CAS: 9005-65-6; a mixture of polyethoxylated derivatives of sorbitan and oleic acid as a non-ionic surfactant) from Sigma-Aldrich (Sintra, Portugal). Ultrapure distilled water was obtained using a Milli-Q^®^ Plus system (Millipore, Darmstadt, Germany). Aucubin (CAS: 479-98-1; chemical formula: (2*S*,3*R*,4*S*,5*S*,6*R*)-2-[-(1*S*,4a*R*,5*S*,7a*S*-5-Hydroxy-7-(hydroxymethyl-1,4a,5,7a-tetrahydrocyclopenta-[*c*]-pyran-1-yl]-oxy]-6-(hydroxymethyl)-oxane-3,4,5-triol) and catalpol (CAS: 2415-24-9; chemical formula: ((2*S*,3*R*,4*S*,5*S*,6*R*)-2-{[(1aS,1bS,2S,5aR,6S,6aS)-6-Hydroxy-1a-(hydroxymethyl)-1a,1b,2,5a,6,6a-hexahydrooxireno[2′,3′:4,5]cyclopenta[1,2-*c*]pyran-2-yl]oxy}-6-(hydroxymethyl)oxane-3,4,5-triol) were used as active substances and were purchased from Angene International Limited (London, UK). HEC (CAS: 9004-62-0; hydroxyethyl cellulose as a gelling agent; Sigma-Aldrich, Poland), glycerol (Chempur, Piekary Śląskie, Poland) and Microcare^®^ SB (CAS: 532-32-1, 24634-61-5; a blend of sodium benzoate and potassium sorbate as a preservative; Thor GmbH, Erfurt, Germany) were used to prepare the hydrogel formulations. Chromatographic analysis was conducted using HPLC water (Avantor Performance Materials, Gliwice, Poland), trifluoroacetic acid (CAS: 76-05-1; Sigma Aldrich, Poznan, Poland) and acetonitrile (CAS: 75-05-8; Avantor Performance Materials, Gliwice, Poland). Potassium dihydrogen phosphate (CAS: 7778-77-0; Merck, Darmstadt, Germany) and sodium hydroxide (CAS: 1310-73-2; Merck, Darmstadt, Germany) were used to carry out the release study.

#### 2.1.2. Skin Testing Equipment

Evaluation of skin condition using non-invasive methods included the use of the following devices: Tewameter^®^ TM 300 (to determine the level of transepidermal water loss, TEWL), Corneometer^®^ CM 825 (skin hydration), Visioscan^®^ VC 98 (skin topography parameters) and Visioline^®^ VL 650 (skin macrorelief parameters). All equipment was purchased from Courage + Khazaka electronic GmbH (Köln, Germany). The importance of technical possibilities of the above-mentioned devices concerning selected skin parameters have been described comprehensively in the authors’ previous publications [21,22].

**Figure 2:**
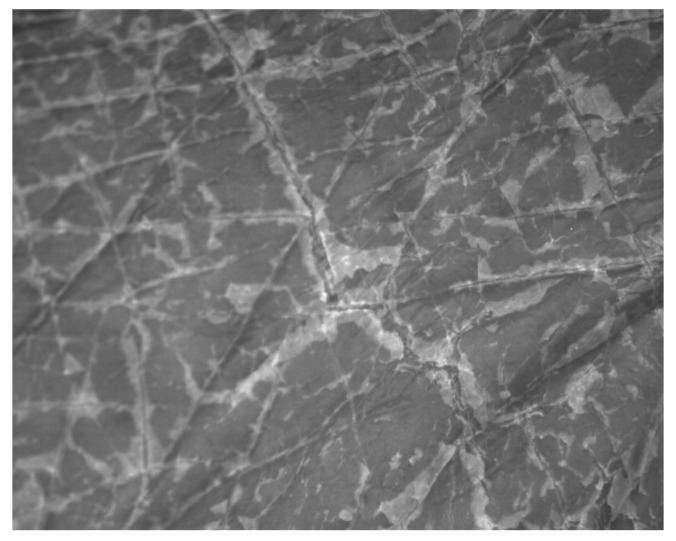
Visioscan^®^ VC 98 image of dehydrated skin; area: right hand, outer side.

**Figure 3:**
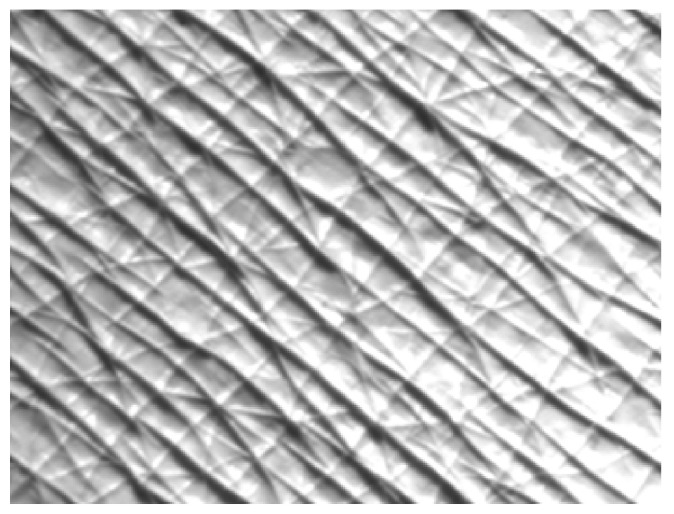
Visioline^®^ VL 650 image of mature skin; area: left forearm, inner side.

The images were obtained during the work using a Visioscan^®^ VC 98 and Visioline^®^ VL 650 and were processed using software specific for these devices. The measurement principle of the Visioscan^®^ VC 98 is based on the analysis of obtained photographs (Figure 2), which are displayed in 256 grey levels (0 value is black and 255 is white). Bright grey colors correspond to dehydrated skin, medium grey levels are associated with proper condition of the skin with a good hydration level, whereas dark grey levels are related to skin furrows and wrinkles, which appear in mature skin [23,24]. Regarding the images obtained by the Visioline^®^ VL 650, the device follows the profilometry principles, which comprise the illumination of a silicone replica of the selected skin area at an angle of 35°. The skin wrinkles (present in the replica) produce measurable shadows, which are then digitalized (Figure 3) and calculated using software [25,26].

#### 2.1.3. Testing Panel

The testing panel consisted of two groups of 25 female volunteers, under 30 years of age (selection criteria: normal skin without any dermatological skin lesions). The pre-test schedule comprised: (i) step one, signing the informed consent form for participation in the study and (ii) step two, the volunteers participated in a dermatological interview, followed by filling out a questionnaire about their health, skin condition, skin care habits and possible skin problems. The in vivo study was approved by the Bioethical Commission of Poznan University of Medical Sciences, Poland, on 1 February 2018 (141/17).

### 2.2. Methods

#### 2.2.1. Production of Lipid Nanoparticles

Lipid nanoparticles were produced using a modified emulsification–ultrasonication method based on multiple emulsion (W/O/W) [27,28]. In brief, the components of lipid phase (Softisan^®^ 100—4.5 wt.%, glycerol—37.5 wt.% and CTAB—0.5 wt.%) and a small amount of Milli-Q^®^ Plus water were heated to 50 °C in separate beakers. Then, the aqueous phase prepared in this way was added to the lipid phase, vigorous stirring of the resulting emulsion was applied using a high-speed Ultra-Turrax^®^ T25 Digital Homogenizer for 5 min at 24,000 rpm (Ystral GmbH, Ballrechten-Dottingen, Germany). The internal W/O emulsion was then homogenized using an Ultrasonic Processor VC130 homogenizer (Sonics, Newtown, CT, USA) for 30 s at a 40% amplitude. The next step was to prepare an aqueous solution of Tween^®^ 80 (1 wt.%; 50 °C) and add it in appropriate amounts to the previously obtained W/O emulsion. The resulting dispersion was homogenized for 90 s at an amplitude of 40%. Then, the remaining part of the aqueous surfactant solution was added to the formed dispersion and stirred using a magnetic stirrer at 300–350 rpm, until the system cooled down. Aucubin and catalpol were incorporated separately into lipid nanoparticles at the stage of the preparation of internal W/O emulsion (in the amount of 0.1 wt.% and 0.09 wt.%, respectively).

#### 2.2.2. Physicochemical Characterization of Lipid Nanoparticles

Mean particle size (Z-Ave), polydispersity index (PDI), and zeta potential (ZP) were determined with a Zetasizer Nano ZS (Malvern Instruments, Malvern, UK). The measurements were preceded by the preparation of aqueous solutions of lipid nanoparticles (400 µL of dispersion in 100 mL of distilled water; pH of solutions equal to approximately 6.0). For each tested sample the procedure was repeated three times. The arithmetic mean and standard deviation were calculated from the obtained results. The encapsulation efficiency of iridoid glycosides in the obtained lipid nanoparticles was determined using high-performance liquid chromatography (Varian 920-LC, Agilent Technologies, Santa Clara, CA, USA) by quantifying the content of non-incorporated active substance present in the external aqueous phase of the surfactant.

#### 2.2.3. Preparation of Hydrogel Formulations

**Figure 4:**
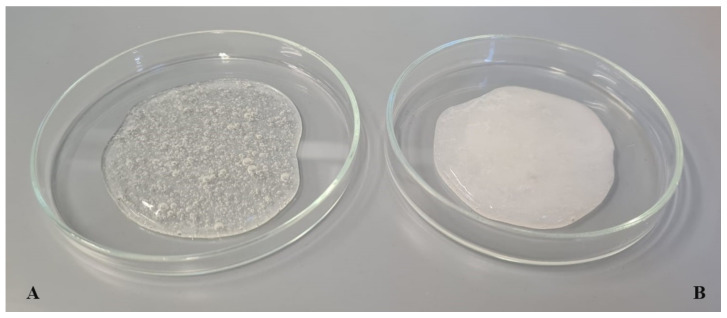
The visual appearance of hydrogel formulation without active ingredients (**A**)**.** Lipid nanoparticle-containing hydrogel preparation (**B**).

Glycerol (10 wt.%) and demineralized water were weighed into a glass beaker and placed in a water bath where they were heated to 80 °C. Hydroxyethyl cellulose (2.5 wt.%) was then gradually added, with vigorous stirring of the resulting formulation until a gel consistency was obtained. After cooling the system to 40 °C, Microcare^®^ SB (0.2 wt.%) was added and the mixture was stirred for 5 min (hydrogel 1, Figure 4A). In the case of hydrogels containing iridoid glycosides (0.5 wt.%) in the traditional form, the next step was to add them to the formulation at a temperature below 30 °C (hydrogels 2 and 3). Hydrogels 4–6 were obtained by introducing the dispersions of lipid nanoparticles (without active ingredients or with aucubin/catalpol) into hydrogel formulations. The hydrogel, previously prepared according to the above procedure, was weighed into a glass beaker. Then, the lipid nanoparticle dispersion (in a ratio of 50:50 wt.%) was added, upon intensive stirring until the desired consistency was obtained. The resulting cosmetic formulation was stirred for another 10 min to stabilize the consistency (Figure 4B).

Detailed compositions of the hydrogel formulations investigated are shown in Table 1.

#### 2.2.4. Physicochemical Characterization of Hydrogel Formulations

The physicochemical parameters (pH, viscosity, and particle size distribution using a laser diffraction technique) were determined. The pH (EcoSense^®^ pH 10 pH/Temperature Meter, Pen Style; VWR International, Radnor, PA, USA) and viscosity (rotational viscometer Rheotec RC02; Merazet, Poznan, Poland) of the hydrogels were tested on the day of preparation of the cosmetic formulation (day 0) and after 60 days (day 60) at room temperature. The particle size distributions (Mastersizer 2000, Malvern Instruments, Malvern, UK) were also measured after 15 (day 15) and 30 days (day 30). The measurement principle of particle size distribution analysis is that a portion of 0.1 g of hydrogel formulation is dispersed in 800 mL of distilled water at a stirring rate of 1500 rpm, maintaining the obscuration values set at the assumed level of 5%. The results were displayed as particle size distribution curves.

Physicochemical characterization was conducted for the hydrogel formulations stored at three temperatures (4, 25, and 40 °C). For each tested sample the procedure was repeated three times. The arithmetic mean and standard deviation were calculated from the results obtained.

#### 2.2.5. Determination of AI in the Hydrogel Formulations (HPLC)

The determination of iridoid glycosides in the hydrogel formulations was carried out using the calibration curve method using a high-performance liquid chromatograph (Varian 920-LC). The chromatographic analysis was performed under the following conditions: (i) column: Zorbax SB-C18 (250 × 4.6 mm; 50 μm); (ii) isocratic mobile phase contained acetonitrile (3%) and 0.1% trifluoroacetic acid in water (97%), delivered at 1 mL/min; (iii) sample volume: 20 µL; (iv) analysis time: 13 min; (v) detector: UV-Vis; (vi) analytical wavelength: 210 nm. Sample preparation consisted in weighing 0.1 g of the hydrogel, adding 10 mL of distilled water, and shaking the sample intensively for about 5 min. The obtained solutions were filtered through syringe filters with a pore diameter of 0.45 μm and 1.5 mL were transferred to glass vials for HPLC analysis. For each tested sample the procedure was repeated three times. The arithmetic mean and standard deviation were calculated from the results obtained.

#### 2.2.6. Release Study

A study of the release efficiency of iridoid glycosides from the hydrogel formulations was carried out using a 708-DS Dissolution Apparatus (Agilent Technologies, Santa Clara CA, USA) in combination with a high-performance liquid chromatograph Varian 920-LC. The analysis was performed under the following conditions: (i) medium: phosphate buffer (pH = 5.8); (ii) membrane: Cuprophan membrane (11.5 µm); (iii) temperature of water bath: 32.0 ± 0.5 °C; (iv) spindle speed: 100 rpm; (v) analysis time: 24 h. The procedure consisted in collecting 1 mL of acceptor fluid from a glass vessel from above the extraction chamber containing the tested hydrogel formulation. Samples were then filtered through syringe filters with a pore diameter of 0.45 μm and transferred to glass vials for HPLC analysis. Measurements were performed every 30 min until the 6th hour of the release study and then at hourly intervals. The subsequent chromatographic analysis was performed, as described in Section 2.2.5. Finally, the calculated concentrations of the iridoid glycosides were converted into the percentage value of their release from the tested hydrogel formulation in relation to their total content in a given cosmetic product.

#### 2.2.7. The Application Tests

The evaluation of hydrogel formulation efficacy included two 4-week in vivo studies, each conducted on a group of 25 female volunteers, who received three hydrogel formulations for testing, which are characterized in detail in Table 2.

The volunteers were provided with information materials regarding the in vivo study (purpose, schedule, application rules, skin care rules). Additionally, the participants were verbally informed about the appropriate application of the tested hydrogel formulations—once a day, on designated areas of the inner side of the left forearm. They also received a template indicating the precise place of hydrogel application. Evaluation of skin condition was based on the measurement of selected skin parameters using non-invasive skin testing equipment (see Section 2.1.2). TEWL and skin moisture levels were measured at weekly intervals, while skin topography and macrorelief parameters were measured at week 0 (before the first application) and at the end of the study (week 4). Statistical analysis was carried out using the Wilcoxon test for pairs of observation results. The level of significance was *p* < 0.05.

## 3. Results and Discussion

### 3.1. Characterization of Lipid Nanoparticles

The optimization of the composition of lipid nanoparticle dispersions is needed prior to industrial-scale synthesis. This is particularly important when planning the composition of lipid nanoparticles containing active substances of a non-lipophilic nature or whose incorporation has not been described in previous studies [27,29,30]. The stability of lipid nanoparticle dispersions significantly affects the effectiveness of the active substances contained in the lipid matrix; should they be introduced into a cosmetic product, they should be stable for at least 30 days [31,32]. Clarifying the issue of incorporation of lipid nanoparticles into cosmetic formulations intended for skin application, it is assumed that cosmetic preparations obtained in this way will be qualified as nanocosmetics only when the average size of the incorporated lipid nanoparticles does not exceed 100 nm [33,34].

The optimum quantitative composition of the lipid nanoparticle dispersion was determined as Softisan^®^ 100 (4.5 wt.%): Tween^®^ 80 (1.0 wt.%) (in the course of carrying out 32 factorial design). The following results were obtained for this lipid nanoparticle dispersion: (i) Z-Ave = 93.32 ± 0.32 nm; (ii) PDI = 0.226 ± 0.004; (iii) ZP = 39.30 ± 4.22 mV. Subsequent incorporation of iridoid glycosides confirmed the appropriate choice of the method of lipid nanoparticle production (emulsification–ultrasonication method based on multiple emulsions). The encapsulation efficiency was determined at the levels of 89% and 77% for aucubin and catalpol, respectively. A directly proportional relationship was observed between the encapsulation efficiency and the degree of hydrophilicity of active compound incorporated into the lipid nanoparticles. Aucubin, an active substance with a higher water solubility, was incorporated to a higher extent into the internal W/O emulsion in the lipid nanoparticle matrix. Bearing in mind that, for lipophilic substances, encapsulation efficiency is in the range of 90–98%, hydrophilic compounds (such as, in this case, aucubin and catalpol) usually achieve relatively lower values due to the poor affinity to the lipid matrix [5,35,36]—the obtained results reached 90% (aucubin) and 80% (catalpol) and can be considered as satisfactory. The obtained lipid nanoparticles incorporated with iridoid glycosides were also characterized by their physicochemical parameters (Z-Ave, PDI, ZP) with the following expected values: aucubin: 84.35 ± 1.84 nm, 0.240 ± 0.007, 47.30 ± 1.47 mV and catalpol: 84.79 ± 1.32 nm, 0.240 ± 0.014, 55.53 ± 1.55 mV, respectively. In conclusion, lipid nanocarriers with a mean particle size below 100 nm, suitable for use as components of cosmetic formulations, were produced.

### 3.2. Physicochemical Characteristics of Hydrogel Formulations

#### 3.2.1. pH Test

The use of skin care products must not cause disturbances to the acid–base balance in individual layers of the skin, and in particular it should not impair the barrier function of the stratum corneum. Consequently, it is important that the pH value of the designed cosmetic formulations should be within the optimal range of 6–7.7 [37]. Table 3 presents the changes in pH values determined for the tested hydrogel formulations over a period of 60 days.

Testing the pH of the obtained hydrogels confirmed their appropriate reaction (borderline between slightly acidic and neutral pH), and all the values were within the optimal range from 6.03 ± 0.02 to 6.40 ± 0.02 during the whole 60-day study period. A decrease in pH value was observed as a result of the addition of iridoid glycosides to the tested cosmetic preparations, but its value was still within the normal range. The method of introduction of the iridoid glycosides (as components of the aqueous phase—formulations 2 and 3 or incorporated into lipid nanoparticles—formulations 5 and 6) had no effect on the degree of decrease in pH value (by about 2%) in relation to that of the cosmetic base (formulation 1). Similar conclusions have been reported by Joshi and Patravale [38], from their studies on NLCs incorporation into gel formulations. Additionally, no correlation was observed between the degree of pH decrease and the specific iridoid glycoside (aucubin or catalpol). The study also did not show a dependence of pH value changes on the storage temperature of the samples (both for the cosmetic base alone and the cosmetic base containing the active substance) and the way the iridoid glycoside was introduced into the cosmetic formulation. The pH value did not fluctuate significantly over time and this was also confirmed by Gao et al., who introduced lornoxicam-loaded NLC into gels based on hydroxypropyl methylcellulose [39].

#### 3.2.2. Viscosity Study

The ease of application of a cosmetic formulation on the skin is determined by the consistency of the finished cosmetic product, which is strictly related to its viscosity [40]. The stability of a cosmetic preparation during storage time, taking into account the viscosity, is related to the lack of a tendency to exhibit instability changes in the nature of particle migration [41].

**Figure 5:**
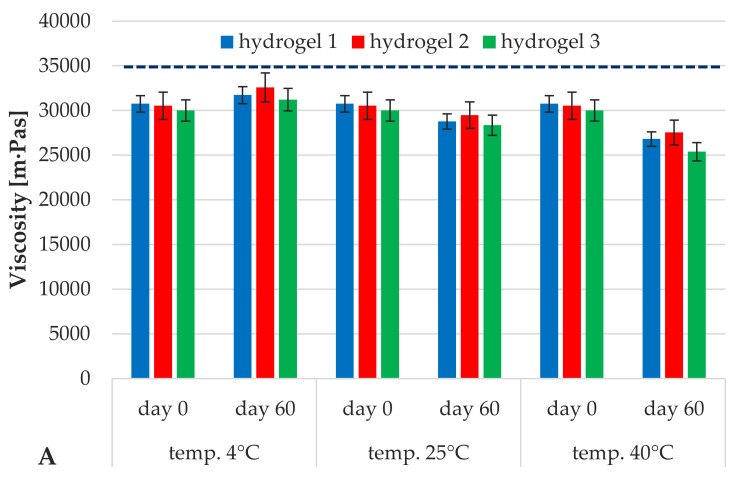

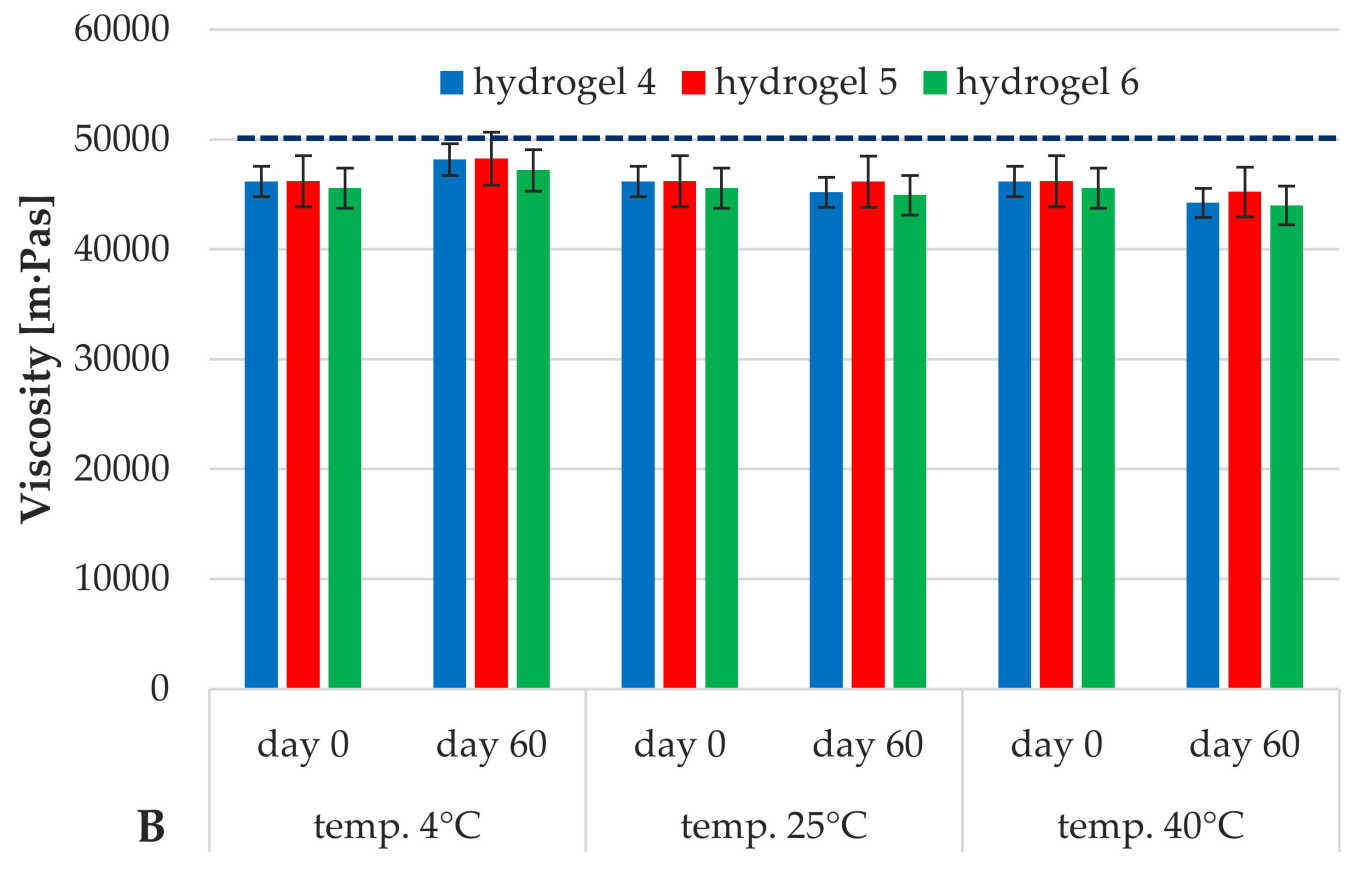
Changes in viscosity of the hydrogel formulations: 1–3 (**A**) and 4–6 (**B**) stored at different temperatures (4, 25, 40 °C) for 60 days; spindle R4; velocity 5 rpm.

In order to illustrate the viscosity results in a more accessible manner, they were divided into two parts: Figure 5A shows the viscosity changes of hydrogel formulations 1–3 containing iridoid glycosides placed in the aqueous phase, while Figure 5B illustrates the analogous changes in viscosity of hydrogels 4–6 enriched with lipid nanoparticles.

According to Figure 5A, the changes in viscosity of the investigated hydrogel formulations (1–3) during the 60-day study period were insignificant. In addition, no effect of storage temperature and addition of iridoid glycoside on changes in viscosity values of the tested hydrogels was observed within 60 days. The recorded changes in viscosity over that time were at a level not exceeding 7%. As shown in Figure 5B, the mean viscosity of hydrogels containing lipid nanoparticles increased significantly, (blue line) with respect to that of the hydrogels containing iridoid glycosides dissolved in water phase, Figure 5A. An over 40% increase was recorded for the formulation with lipid nanoparticles (hydrogels 4–6), which means that this cosmetic product was characterized with a texture and consistency more attractive to potential users. The results correspond to those obtained by other research groups, e.g., Joshi [38] and Junyaprasert [42] indicated the stabilizing effect of lipid nanoparticles on the viscosity of the hydrogels they studied. The same authors have noted a similar trend in changes in viscosity values over time, as in the case of hydrogels 1–3, independent of the storage temperature of the samples. However, the intensity of the changes was significantly lower (around 2%) in comparison with that for hydrogels not containing lipid nanoparticles. An increase in the content of lipid raw materials (forming the matrix of lipid nanoparticles) was determined as the factor influencing the growth and lower fluctuations of viscosity of hydrogels containing them. Thus, the introduction of lipid nanoparticles into hydrogel formulations was identified as the key factor in stabilizing the consistency of the cosmetic preparations studied.

#### 3.2.3. Particle Size Distribution Analysis

Particle size distribution (PSD) is a crucial parameter of physicochemical characterization and stability of cosmetic formulations, determining the degree of homogeneity of a designed cosmetic product [43,44]. PSD analysis enables identification of possible phenomena of instabilities appearing in the sample, with particular consideration of those unnoticeable to the human eye, and connected with gradual agglomeration of cosmetic formulation particles [45,46].

Analysis of the particle size distribution results obtained for the hydrogel formulations investigated was based primarily on the observation of changes in the shape of the particle size distribution curves and in the value of the parameter d(0.9) (Table 4), determined for hydrogel samples stored under different temperature conditions (4, 25 and 40 °C) for 60 days.

**Figure 6:**
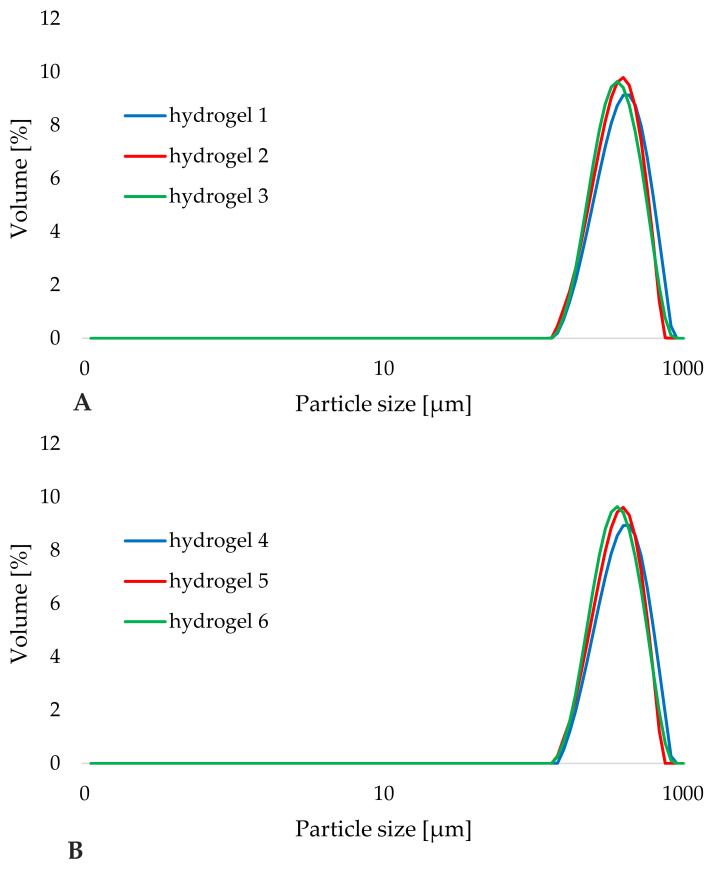
Comparison of particle size distribution curves of the hydrogel formulations: 1–3 (**A**) and 4–6 (**B**) on the day of preparation (day 0).

The interpretation of the PSD results began by determining the effect of the presence of the active substance (iridoid glycosides) on the shape of the particle size distribution curves of the hydrogel formulations containing it. Figure 6 shows the PSD curves obtained for the investigated hydrogels: cosmetic base without the active compound (blue) and hydrogels enriched with aucubin (red) and catalpol (green) on the day of their production. No visible changes in the shape of the particle size distribution curves and the value of the d(0.9) parameter (Table 4) were determined as a result of the introduction of iridoid glycosides to all the tested hydrogel formulations. The curves shown in Figure 6A,B were almost overlapping. Moreover, the method of introduction of aucubin and catalpol (as components of the aqueous phase or in the form of lipid nanoparticles) did not affect the particle size distribution and the level of the d(0.9) parameter, in the measuring range of the device (0.2–2000 μm). Unfortunately, this limitation made it impossible to detect structures in the form of lipid nanoparticles of sizes smaller than 100 nm. Preliminary PSD analysis performed on the day of hydrogel production indicated the compatibility of added iridoid glycosides with the remaining components of the hydrogel preparations.

**Figure 7:**
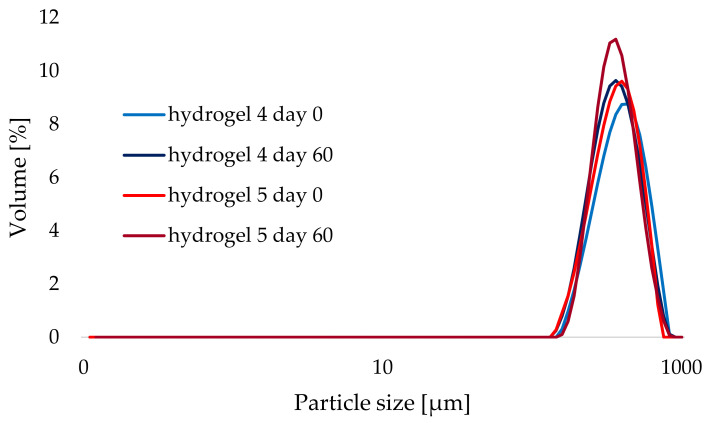
Comparison of particle size distribution of hydrogels 4 and 5 stored at 25 °C (day 0 and day 60).

**Figure 8:**
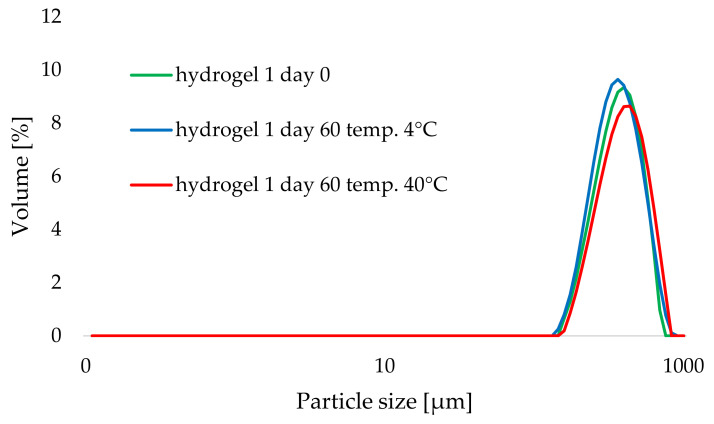
Comparison of particle size distribution of hydrogel 1 stored at 4 and 40 °C (day 0 and day 60).

An attempt was made to determine the effect of temperature and storage time on the stability of the hydrogel formulations studied. Figure 7 and Figure 8 show the effects of storage time (comparison of hydrogel 4 as a cosmetic base with “empty” lipid nanoparticles and hydrogel 5 containing aucubin-loaded lipid nanoparticles, stored at 25 °C for 60 days) and temperature (comparison of PSD curves obtained for hydrogel 1, stored at 4 and 40 °C for 60 days) on the particle size distribution of the tested hydrogel formulations. As in the previous analysis, no changes were observed in the particle size distribution curves, and the resulting curves almost overlapped each other. When it comes to the parameter d(0.9), the minimum fluctuations of its value were caused by elevated temperature and the 60-day storage period (see the red curves in the graphs in Figure 7 and Figure 8, also Table 4). Hence, sufficient homogeneity of the investigated hydrogel formulations was confirmed using laser diffraction technique and no effect of lipid nanoparticles incorporation on shelf-life stability of the hydrogels was detected. This observation confirmed the earlier reports by other authors working on the subject—they also obtained stable hydrogels formulations containing lipid nanoparticles, e.g., Souto who investigated hydrogels with different gel-forming agents [31] or Pokharkar who studied formulations enriched with eugenol-loaded nanostructured lipid carriers [47].

### 3.3. Evaluation of Chemical Stability of Iridoid Glycosides in the Hydrogel Formulations (HPLC)

Chemical stability of an active ingredient in a cosmetic product depends on its compatibility with the remaining components of the cosmetic preparation, as well as on the time and storage conditions of the sample [48]. Chemical stability significantly affects the degree of release of the active compound from a cosmetic base, and thus the effective action of the cosmetic formulation [49].

**Figure 9:**
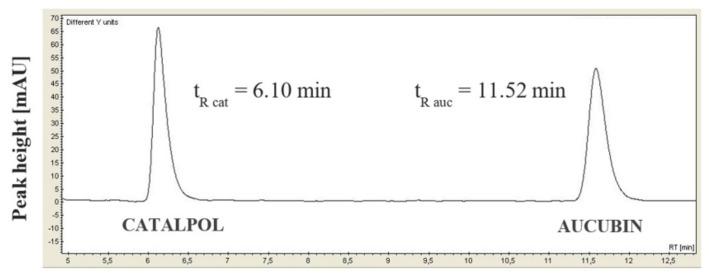
Chromatogram of standard solution of aucubin (50 μg/mL) and catalpol (50 μg/mL).

In the present work, we developed a methodology for qualitative and quantitative determination of iridoid glycosides in the hydrogel formulations using high-performance liquid chromatography. On the basis of the chromatograms obtained, the retention times of iridoid glycosides—catalpol and aucubin—were determined to be 6.10 min and 11.52 min, respectively (Figure 9). Moreover, in order to determine the possible influence of other components on the result of chromatographic analysis, the samples of hydrogel formulations not containing iridoid glycosides (hydrogels 1 and 4) were also examined. The chromatograms obtained did not reveal the presence of peaks originating from other formulation components in the range of aucubin and catalpol retention times (5–13 min).

As far as the quantitative analysis is concerned, the calibration curves for aucubin and catalpol were prepared and the linear regression equations (aucubin: y = 0.3487x − 0.2196; catalpol: y = 1.0846x + 12.233) and the correlation coefficients R2 were determined. The values of R2 were 0.9993 and 0.9974 for aucubin and catalpol, respectively, falling within the recommended range of values of 0.9–1.0, indicating a wide range of linearity of the method developed. In addition, the high repeatability of the method was determined by the standard deviation values obtained. The quantitative analysis was performed in order to check for possible changes in the concentration of iridoid glycosides in the hydrogel preparations, which may occur as a result of progressive instability of the active ingredient contained in the cosmetic preparation. Results of the quantitative analysis are collected in Table 5, which presents concentration values of iridoid glycosides, calculated on the basis of linear regression equations. For a clearer presentation of changes in the content of iridoid glycosides in the hydrogel products, in addition to the concentration value of the active compound, they were also presented in the form of the loss of the active substance (expressed in%) in 60 days in relation to its initial content on the day of preparation of the hydrogel samples. As to the amounts of iridoid glycosides added to hydrogels on the day of production, their maximum theoretical concentrations in the sample of the hydrogel formulation prepared for chromatographic analysis were: (i) for aucubin: 50.00 μg/mL (hydrogel 2); 5.00 μg/mL (hydrogel 5); (ii) for catalpol: 50.00 μg/mL (hydrogel 3); 4.50 μg/mL (hydrogel 6).

The results of quantitative analysis indicated satisfactory chemical stability of the iridoid glycosides tested. The loss of the active substance in all tested hydrogel formulations did not exceed 4%. Moreover, no statistically significant difference was found between the chemical stability of aucubin and catalpol—the obtained values and the tendency of changes in the content of iridoid glycosides in the hydrogel formulations showed similar characters. Analyzing the effect of storage temperature of hydrogels on chemical stability of iridoid glycosides, relatively higher stability of aucubin and catalpol was determined in hydrogel preparations stored at 4 °C (1.4 and 1.2%, respectively) when compared to that of the samples exposed to elevated temperature of 40 °C (3.2% for both glycosides). A positive effect of incorporation of iridoid glycosides into lipid nanoparticles on the degree of decrease in their content in all obtained hydrogels was also observed. When comparing the corresponding cosmetic formulations, i.e., hydrogels 2 and 5, as well as hydrogels 3 and 6, differing in the way the active substance was incorporated into the cosmetic base, it appears that in the hydrogels containing lipid nanoparticles, iridoid glycosides were protected by the lipid carrier and showed higher chemical stability compared to that of the samples in which the glycosides were added to hydrogel preparations in a traditional way. This relationship was observed regardless of the storage temperature of the samples. The results reported by Junyaprasert and co-workers [42] support our results, indicating a beneficial influence of incorporation of coenzyme Q10 into lipid nanoparticles on its chemical stability in hydrogel preparations, as well as a negative effect of elevated storage temperature on the loss of active ingredient in the cosmetic product.

### 3.4. Assessment of the Release Profiles of Iridoid Glycosides in the Hydrogels Formulations

**Figure 10:**
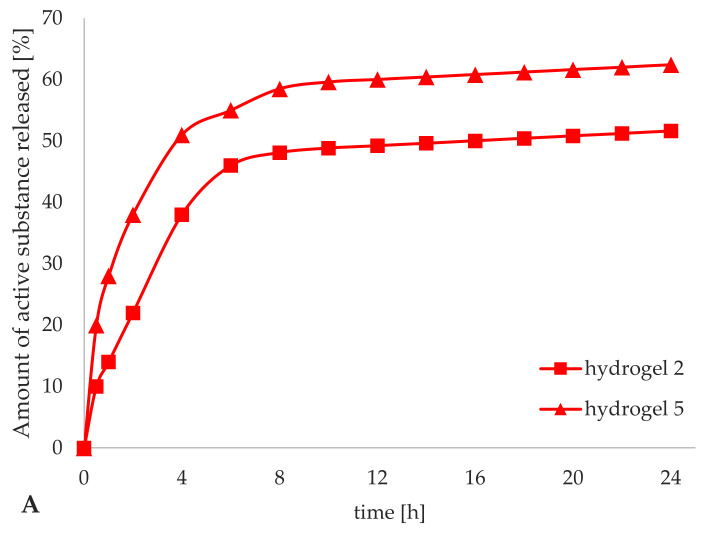

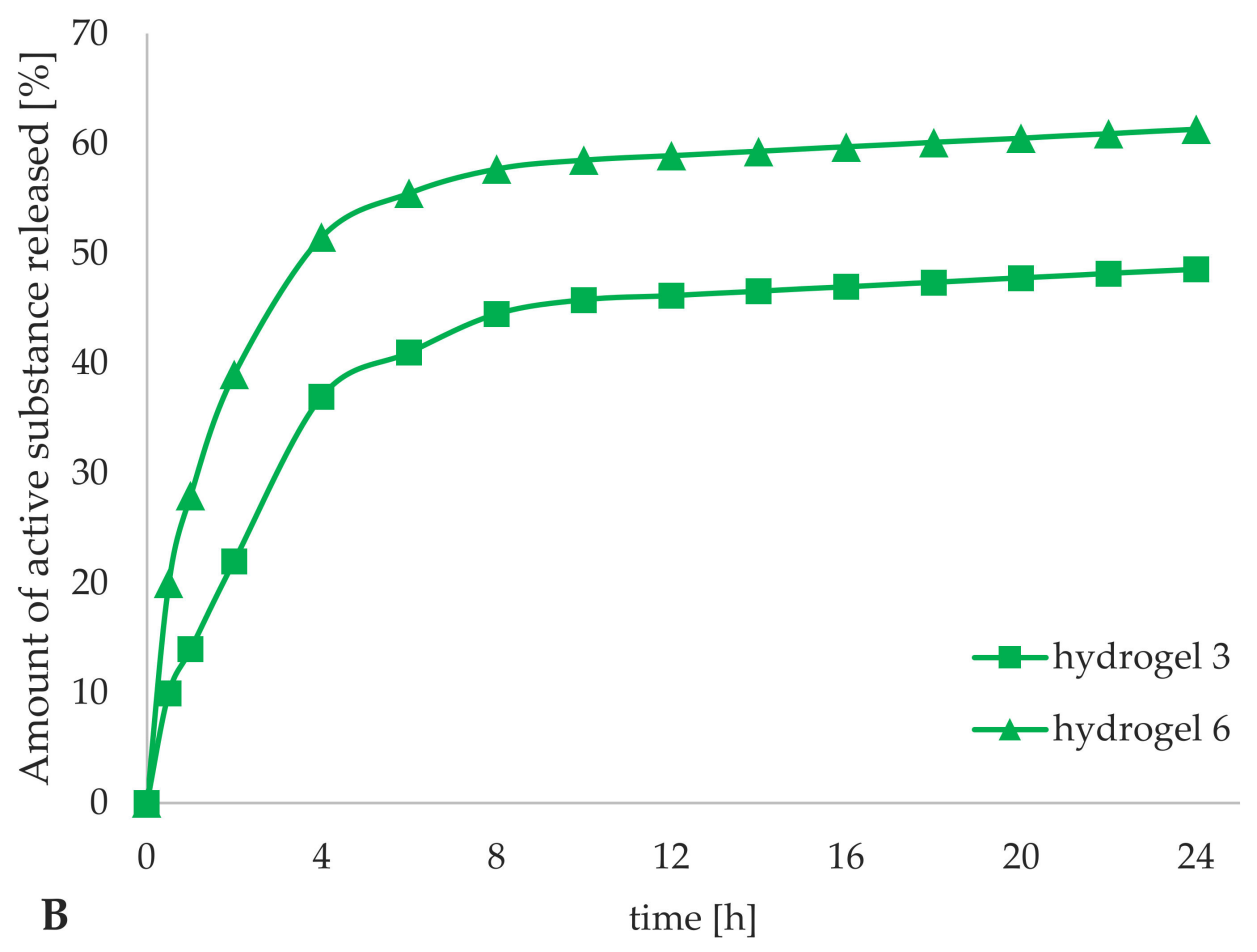
Release profiles of aucubin (**A**) and catalpol (**B**) for the hydrogel formulations (2, 3, 5, and 6).

The release profile of active substance from cosmetic formulations depends to a high degree on the type of cosmetic medium [50,51]. The study of the efficiency of release of iridoid glycosides from hydrogel preparations included an assessment of their ability to penetrate from the hydrogel to the acceptor fluid, where the subsequent change in the concentration of aucubin and catalpol was determined using high-performance liquid chromatography. The amount of released iridoid glycosides was calculated on the basis of the concentrations of aucubin and catalpol in the samples of acceptor fluid, and converted into the percentage value of the degree of release of iridoid glycosides from the hydrogel formulations in relation to their total, theoretical content in the sample of the hydrogel prepared for chromatographic analysis: (i) for aucubin: 22.50 μg/mL (hydrogel 2); 2.50 μg/mL (hydrogel 5); (ii) for catalpol: 22.50 μg/mL (hydrogel 3); 2.25 μg/mL (hydrogel 6). The release profile of iridoid glycosides from hydrogel formulations are presented in Figure 10, as the percentage of released aucubin (Figure 10A) and catalpol (Figure 10B) as a function of time.

The presented release profiles did not show any differences in the degree of release of aucubin and catalpol from all the tested hydrogel formulations and were determined as classical, indicating a gradual release of the iridoid glycosides from the cosmetic base over time. Even for hydrogels 5 and 6 containing iridoid glycosides encapsulated in lipid nanoparticles, no characteristic biphasic release profile was observed due to the method used to obtain lipid nanoparticles, in which the incorporation of active ingredients occurs into the aqueous phase of the internal emulsion. Similar observations have been made by Farboud [32] when studying the release efficiency of coenzyme Q10 (incorporated into SLNs) from O/W cream. The degree of iridoid glycosides release was significantly depended on the form in which they were introduced to the hydrogels; the relevant results were for hydrogels 5 and 6 the release was 62.4% for aucubin vs. 61.3% for catalpol in the form of nanoparticles and for hydrogels 2 and 3 the corresponding results were 51.6% for aucubin vs. 48.6% for catalpol when introduced without the carrier. The mean level of iridoid glycosides release for the hydrogels containing the active ingredients dissolved in water phase (hydrogels 2 and 3) was 50.1%. The reason for such a low level of release was a high hydrophilic character of the support which shows strong affinity to aucubin and catalpol being also of hydrophilic character. Therefore, the hydrogel support could not provide such a high release of the active substance as would be achieved for lipophilic active ingredients. Incorporation of iridoid glycosides into lipid nanoparticles resulted in increased lipophilicity and altered the release of aucubin and catalpol from the cosmetic base to which they were added. Taking into account the results for hydrogel formulations 5 and 6 (containing lipid nanocarriers), the incorporation of lipid nanoparticles changed the nature of hydrogel towards physicochemical form of lipogel (still remaining a hydrophilic substrate). However, the increase in lipophilicity was sufficient to increase the release of iridoid glycosides from hydrogels 5 and 6 by an average of 11.8%, relative to that obtained for hydrogels 2 and 3 (hydrogels containing active substances dissolved in aqueous phase).

### 3.5. Skin Parameters Estimation (In Vivo Tests)

Evaluation of the effectiveness of cosmetic products using non-invasive methods is the final stage of the study conducted in order to verify the declared properties of a cosmetic formulation based on the results of objective measurements of skin parameters [52]. Parameterization of changes occurring within the skin as a result of application of cosmetics is gradually becoming an obligatory requirement for producers of modern cosmetic preparations, especially those containing innovative cosmetic raw materials in the form of lipid carrier systems of active substances [53,54].

In vivo studies (1 and 2, see Section 2.2.7) were designed to evaluate the effectiveness of the hydrogel formulations, including determination of the influence of iridoid glycosides, aucubin and catalpol, and the method of their introduction into the hydrogels on selected skin parameters.

#### 3.5.1. Analysis of the Transepidermal Water Loss and the Skin Hydration Measurement

The level of epidermis hydration and transepidermal water loss are coupled skin parameters determining the ability of the skin to bind and retain water in its structures [55]. The essence of skin moisturizing through application and regular use of cosmetic skin care products is preserved only if the cosmetic used also has the ability to ensure sufficient barrier properties of the epidermis, protecting it against excessive transepidermal water loss [56,57,58].

**Figure 11:**
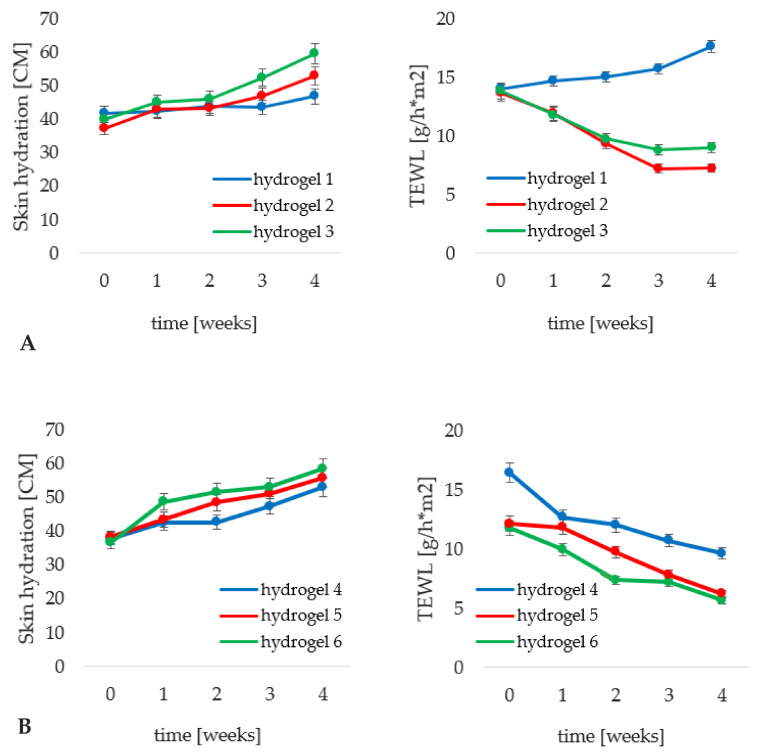
Changes in skin hydration and transepidermal water loss determined for the hydrogel formulations 1–3 (**A**) and 4–6 (**B**) during in vivo studies; *p* < 0.05.

The changes in epidermis hydration and the associated changes in the TEWL level are illustrated in Figure 11. The hydrophilic base without iridoid glycosides (hydrogel 1), was able to increase the epidermis moisture level by 12%, which was accompanied by an increase in the TEWL parameter by over 25%. The reason was the very nature of the cosmetic base which, as a highly hydrophilic substrate, does not possess properties predisposing it to the formation of a protective film on the epidermal surface that would inhibit excessive evaporation of water from the skin structures. However, the addition of iridoid glycosides in the form of aqueous phase components, hydrogels 2 (aucubin) and 3 (catalpol) (Figure 11A) resulted in increasing the epidermis hydration level by 42 and 49%, respectively. In contrast to hydrogel 1, the application of both cosmetic preparations resulted in a decrease in TEWL values, by 47% for the hydrogel with aucubin and by 35% for that with catalpol. The obtained results, concerning the level of epidermis hydration and TEWL, were correlated. The use of the cosmetic product containing catalpol (hydrogel 3) caused an increase in hydration of stratum corneum and reduced the ability of the hydrogel to maintain epidermis barrier function. A more beneficial relation was observed for hydrogel 2, containing aucubin. The reason for less favorable TEWL values obtained for hydrogel 3 is attributed to the fact that catalpol, due to its more hydrating properties, causes a noticeably higher increase in skin hydration after application of the hydrogel containing it to the skin as compared to hydrogel containing aucubin (hydrogel 2). As mentioned above, hydrogel alone, as a substrate without lipid components in its composition, is not able to maintain TEWL at such a high level of stratum corneum hydration.

Comparing the results obtained for hydrogel 1 (cosmetic base) and the formulation containing carrier systems (hydrogel 4) it is apparent that the addition of non-incorporated lipid nanoparticles caused a significantly higher increase in epidermis hydration (by over 40%). Simultaneously, the epidermis barrier function was enhanced as manifested by a decrease in the TEWL level by 41%. It should be remembered that the hydrophilic base itself (hydrogel 1) caused an antagonistic effect. The above relationship was determined by the ability of lipid nanoparticles to gradually regenerate the epidermal barrier by building into the lipid structures of the intercellular matrix of the stratum corneum [59]. Incorporation of iridoid glycosides into lipid nanoparticles resulted in a progressive improvement of the moisturizing effect of the cosmetic products. For hydrogel 5 (Figure 11B), containing aucubin encapsulated in lipid nanoparticles, the increase in the epidermis moisturization (46%) was at a level comparable to that obtained for hydrogel 4; however, the former hydrogel permitted preservation of stratum corneum barrier functions to a greater degree (decrease in TEWL by 52%). Nevertheless, the most noticeable moisturizing effect was observed for hydrogel 6 (enriched with nanoparticles incorporated with catalpol) manifested by 60% increase in moisturization with simultaneous reduction of TEWL level by 49%. A relation between the method of introduction of iridoid glycosides and achieved cosmetic effect was also observed. A superior moisturizing effect was observed for the hydrogel preparations containing lipid nanoparticles incorporated with aucubin and catalpol. They caused an increase in the epidermis hydration level and a decrease in the TEWL value by over 10% greater than the hydrogel with iridoid glycosides introduced in a classical way. Farboud [32] and Junyaprasert [42], have also indicated a noticeable moisturizing effect of cosmetic preparations containing lipid nanoparticles.

#### 3.5.2. Analysis of the Skin Topography Parameters

Skin topography parameters, determined on the basis of the analysis of images of presenting the examined skin area in different shades of grey, provide information on the epidermal surface in terms of the state of homogeneity of its structure and the general condition of the skin, related to the number of skin furrows and the level of hydration of stratum corneum [23,53]. In the course of in vivo studies, it was assumed that the systematic use of skin care products induces the desirable tendency of changes in selected parameters of skin topography as follows:

- decrease in the values of SELS parameters: (i) SEr (roughness) associated with the number of dark pixels present in the image, reflecting wrinkles and furrows in the skin; (ii) SEsm (smoothness) understood as the homogeneity of the obtained image in terms of the lowest possible variation of grey levels in the images; (iii) SEsc (scaliness) expressed as the number of white pixels in the image, identified as the skin with reduced hydration level [21,23,24];
- increase in NRJ (energy) and HOM (homogeneity) values and decrease in CONT (contrast) values, which are specific indicators of the uniformity and overall condition of the skin surface, calculated by analyzing repetitions of combinations of colors of neighboring pixels in the image and the difference between their levels of grey [21,23].

**Figure 12:**
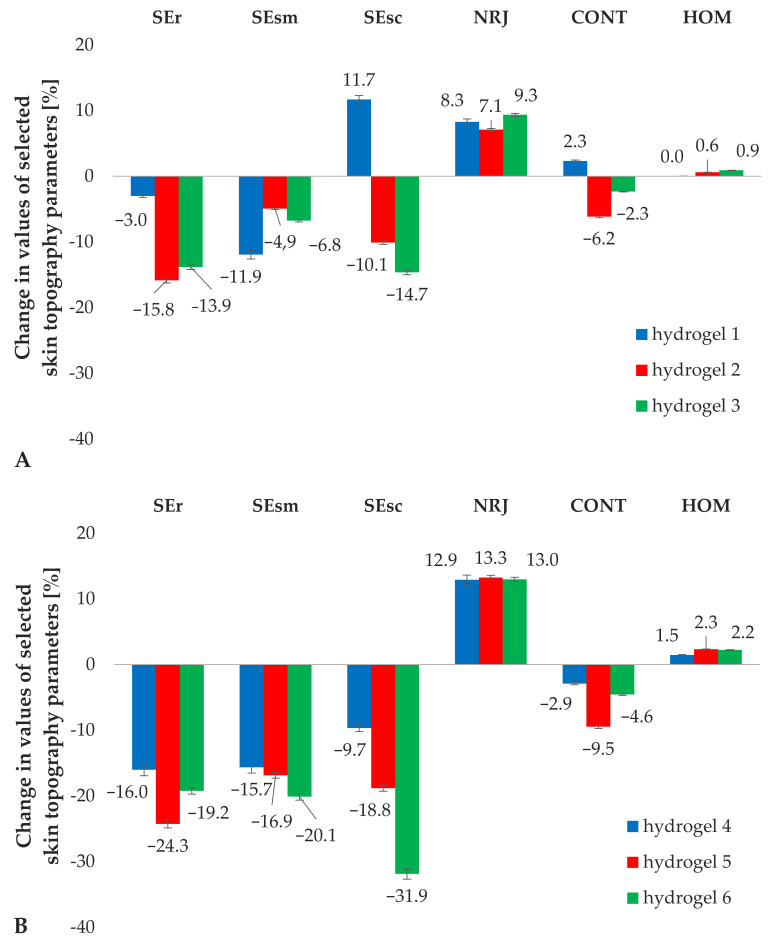
Changes in skin topography parameters determined for the hydrogel formulations 1–3 (**A**) and 4–6 (**B**) during in vivo studies.

Figure 12 shows the averaged changes in selected skin topography parameters (expressed in%), between the data recorded in week 0 and the end of the study, week 4. As follows from the results, the addition of iridoid glycosides (hydrogels 2 and 3) to the cosmetic base (hydrogel 1) brought the expected direction of changes in the SELS parameters (Figure 12A). The regular application of the hydrogel formulations containing aucubin and catalpol decreased SEr level to a greater extent, by 16 and 14%, respectively, in comparison with the use of the hydrogel without the active substance (statistically insignificant decrease by 3%), attributing stronger anti-aging properties to aucubin. A greater degree of reduction in SEsm values was observed for the hydrogels enriched with iridoid glycosides, especially catalpol, indicating its stronger effect ensuring homogeneity of the stratum corneum surface. The hydrogel vehicle (hydrogel 1) caused an undesirable increase in the level of SEsc parameter by 12%, confirming its inability to maintain the correct level of epidermal hydration, as determined in analysis of TEWL results from the previous Section 3.5.1. The beneficial effect of hydrogel formulations containing iridoid glycosides (hydrogels 2 and 3) on the degree of epidermis scaliness was observed by decreasing the SEsc value by 10% and 15%, respectively. On the basis of the relationship between the results of SEsc and the previously described skin hydration, catalpol was identified as an active substance playing a more significant role in the moisturizing effect of the cosmetic product. As far as the texture parameters are concerned, statistically significant changes were obtained only in NRJ parameter, whose level was determined as comparable for all tested hydrogel preparations (1–3).

Comparing the effect of the addition of non-incorporated lipid nanoparticles (hydrogel 4) to that obtained for the hydrogel substrate alone (hydrogel 1), a positive change in SELS and texture parameters was observed. The enrichment of the hydrogel formulation with the lipid nanocarriers caused a favorable difference manifested as a decrease in SEr value by further 13%, SEsm—4% and SEsc—21%, while the hydrogel base (hydrogel 1) caused an undesirable increase in SEsc parameter. A tendency of changes in SELS parameters observed for hydrogels 5 and 6 (Figure 12B) was similar to the fluctuations obtained for the corresponding hydrogels, 2 and 3, indicating a more beneficial effect of the cosmetic products containing aucubin and catalpol in the form of lipid nanoparticles. Aucubin was found to be an anti-ageing active ingredient as the application of hydrogel 5 resulted in further 10% decrease in SEr, while catalpol (encapsulated in lipid carrier—hydrogel 6) was identified as the highly moisturizing active component, reducing the levels of SEsm and SEsc by further 13 and 17%, respectively. Analyzing the results of texture parameters, it was noted that in contrast to the results obtained for hydrogel formulations 1–3, the changes induced by hydrogels containing lipid nanoparticles were found to be statistically significant. The most beneficial changes were a decrease in CONT parameter and an increase in HOM which were greater on average by 3% and 2% for hydrogels 5 and 5 than for the samples with non-incorporated lipid nanoparticles. Analysis of the skin topography parameters indicated a higher efficiency of hydrogels formulations enriched with iridoid glycosides incorporated into lipid nanoparticles. The anti-wrinkle ability of aucubin manifested as a decrease in SEr and intensive moisturizing properties of catalpol in the mechanism of reduction of SEsm and SEsc values were evidenced.

#### 3.5.3. Analysis of the Skin Macrorelief Parameters

Objective analysis of the skin macrorelief and related parameters enables evaluation of the skin structure paying particular attention to the presence of various types of wrinkles and furrows, observed in the course of appropriate illumination of silicone replicas of the skin [25,26]. It was assumed in the in vivo study that the anti-wrinkle effect is parametrically observed as a decrease in the values of the skin macrorelief parameters (total wrinkle area, mean length and depth of the wrinkles) [21,25].

**Figure 13:**
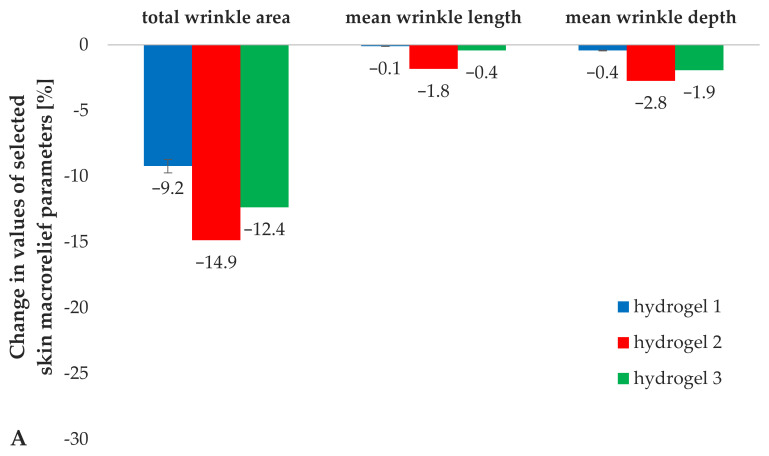

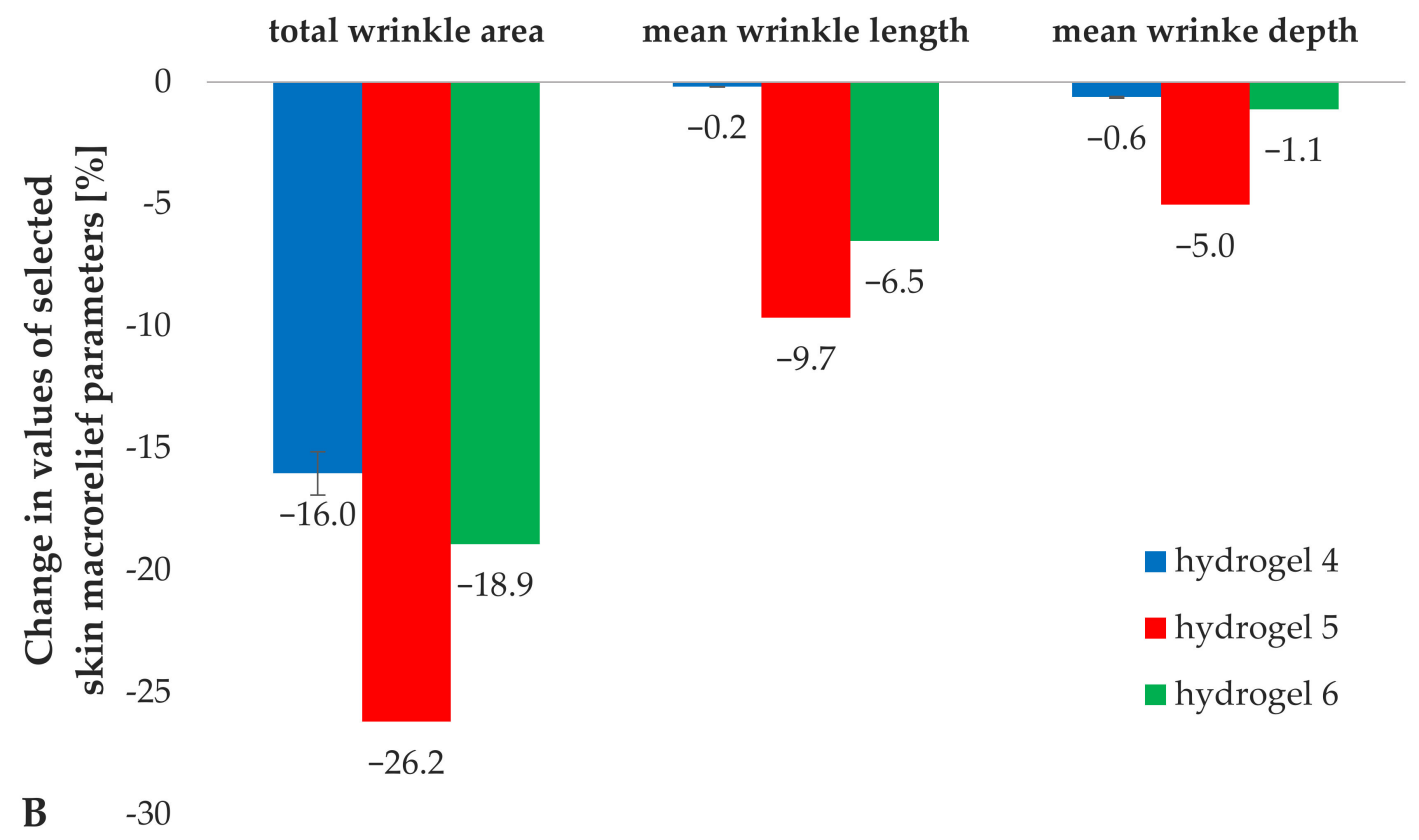
Changes in skin macrorelief parameters determined for hydrogel formulations 1–3 (**A**) and 4–6 (**B**) during in vivo studies.

Analysis of the skin macrorelief parameters (Figure 13) indicated a positive effect of the addition of iridoid glycosides to the hydrogel medium (hydrogel 1). Hydrogels 2 and 3, containing aucubin and catalpol, respectively, reduced the value of skin macrorelief parameters by more than 4% on average in the total wrinkle area and by 1 and 2%, in the average length and depth of the wrinkles, relative to the results obtained for hydrogel 1, which did not contain the active substances (Figure 13A).

The incorporation of lipid nanoparticles into a hydrogel support (comparison of results for hydrogel 1 and 4) resulted in an increase in the reduction of the total wrinkle area by over 7%, again confirming the positive effect of lipid nanoparticles on skin condition, i.e., macrorelief parameters, reduction of the visibility of wrinkles and furrows. No statistically significant changes were determined in the length and depth of the wrinkles on addition of non-loaded lipid nanocarriers to the hydrogel formulation (Figure 13B). A beneficial effect of the hydrogels containing incorporated iridoid glycosides was noted in terms of skin macrorelief parameters. The application of hydrogel formulations enriched with the incorporated lipid nanoparticles reduced the total wrinkle area by an average of 7% relative to the value obtained for hydrogel 4 (containing lipid nanoparticles without active substances). Similar relations were determined for the average length and depth of wrinkles, the application of hydrogels 5 and 6 resulted in a decrease by an average of 8% and 2% relative to the values obtained for hydrogel 4. Considering the relation between the mode of introduction of an active substance into a hydrogel preparation and its effectiveness, a higher degree of wrinkle visibility reduction was observed for hydrogels containing iridoid glycosides in the form of lipid nanoparticles. Comparing the corresponding cosmetic products containing aucubin (hydrogels 2 and 5) and catalpol (hydrogels 3 and 6), a progressive decrease in the values of the skin macrorelief parameters by 9% (total wrinkle area), 7% (mean wrinkle length) and 1% (mean wrinkle depth) on average, was observed. Moreover, it should also be mentioned that the analysis of skin macrorelief parameters clearly indicated aucubin as the active substance with the greatest influence on the parameters related to the presence of wrinkles and furrows, which highly correlated with the results obtained during the analysis of skin topography parameters, which indicated that aucubin shows strong anti-wrinkle properties. Compared to the other tested hydrogel formulations, the cosmetics containing aucubin as an active ingredient caused a decrease in the values of all skin macrorelief parameters, by 10% in the total wrinkle area, and by 6 and 4% in the average length and depth of the wrinkles, respectively.

## 4. Conclusions

Aucubin and catalpol (belonging to the group of iridoid glycosides) have been tested as effective cosmetic raw materials in hydrogel formulations. Incorporation of active compounds into hydrogels was carried out both in a traditional way (aqueous phase ingredients) and by encapsulation into lipid nanoparticles. A correlation between the method of incorporation of iridoid glycosides and the changes in physicochemical parameters of hydrogel formulations within the study was observed. The lipid nanoparticles have been identified as components that favorably influence the physicochemical characteristics of hydrogels within the range of determined parameters (viscosity, particle size distribution and chemical stability of iridoid glycosides). A higher level of release of aucubin and catalpol from the hydrogels containing iridoid glycosides-loaded lipid nanoparticles was noticed. Finally, in vivo study was undertaken to verify the potential for using optimized lipid nanoparticles incorporated with aucubin and catalpol in hydrogel preparations. The synergistic effect of iridoid glycosides and lipid nanocarriers on hydrating and anti-aging effect was evidenced. The specific role of aucubin as an anti-wrinkle ingredient through its ability to effectively reduce SEr and skin macrorelief parameters was indicated. As regards catalpol, it was identified as a raw material with intensive moisturizing properties, manifested by reduction of SEsm and SEsc values. The results obtained in this study have demonstrated higher efficiency of hydrogel formulations enriched with active compounds incorporated into lipid nanoparticles.

## Figures and Tables

**Table 1:** Compositions of hydrogel formulations prepared within the framework of the study.

HYDROGEL FORMULATION	INGREDIENT	QUANTITY [WT.%, ±0.01]
HYDROGEL 1	Hydroxyethyl cellulose	2.50
	Glycerol	10.00
	Microcare^®^ SB ^1^	0.20
	Demineralized water	87.30
HYDROGEL 2 * HYDROGEL 3 **	Hydroxyethyl cellulose	2.50
	Glycerol	10.00
	Microcare^®^ SB ^1^	0.20
	Demineralized water	86.80
	Active substance (aucubin */catalpol **)	0.50
HYDROGEL 4 *** HYDROGEL 5 **** HYDROGEL 6 *****	Hydroxyethyl cellulose	1.25
	Glycerol	5.00
	Microcare^®^ SB ^1^	0.10
	Demineralized water	43.65
	Lipid nanoparticle dispersion (without active ingredients ***/ with aucubin ****/catalpol *****)	50.00 ^2^

^1^ Microcare® SB (a blend of sodium benzoate and potassium sorbate); ^2^ Solid lipids represent 2.25 wt.% of the hydrogel preparation; a single “*” is a hydrogel with aucubin; “**” is a hydrogel with catalpol; “***” a hydrogel with “empty” nanoparticles; “****” is a hydrogel with aucubin-loaded lipid nanoparticles; “*****” is a hydrogel with catalpol-loaded lipid nanoparticles.

**Table 2:** List of hydrogel formulations tested within the framework of the in vivo study.

STUDY NUMBER	COSMETIC NUMBER	CHARACTERISTIC OF HYDROGEL FORMULATION
1	HYDROGEL 1	hydrogel without iridoid glycosides
	HYDROGEL 2	aucubin-containing hydrogel
	HYDROGEL 3	catalpol-containing hydrogel
2	HYDROGEL 4	hydrogels enriched with lipid nanoparticles without iridoid glycosides
	HYDROGEL 5	hydrogels enriched with aucubin-loaded lipid nanoparticles
	HYDROGEL 6	hydrogels enriched with catalpol-loaded lipid nanoparticles

**Table 3:** Changes in pH of the hydrogel formulations stored under various temperature conditions (4, 25, 40 °C) for 60 days.

	pH [-, ±SD]					
	Temp. 4 °C		Temp. 25 °C		Temp. 40 °C	
	Day 0	Day 60	Day 0	Day 60	Day 0	Day 60
HYDROGEL 1	6.36 ± 0.02	6.40 ± 0.02	6.36 ± 0.02	6.40 ± 0.02	6.36 ± 0.02	6.36 ± 0.01
HYDROGEL 2	6.26 ± 0.01	6.30 ± 0.01	6.26 ± 0.01	6.11 ± 0.02	6.26 ± 0.01	6.16 ± 0.01
HYDROGEL 3	6.20 ± 0.01	6.03 ± 0.02	6.20 ± 0.01	6.06 ± 0.01	6.20 ± 0.01	6.17 ± 0.02
HYDROGEL 4	6.26 ± 0.02	6.31 ± 0.01	6.26 ± 0.01	6.32 ± 0.02	6.26 ± 0.02	6.24 ± 0.01
HYDROGEL 5	6.14 ± 0.01	6.18 ± 0.01	6.14 ± 0.01	6.12 ± 0.02	6.14 ± 0.01	6.11 ± 0.01
HYDROGEL 6	6.12 ± 0.02	6.07 ± 0.02	6.12 ± 0.02	6.10 ± 0.01	6.12 ± 0.02	6.11 ± 0.03

**Table 4:** Changes in the value of the parameter d(0.9) determined for the hydrogel formulations stored in various temperature conditions (4, 25, 40 °C) for 60 days.

		d(0.9) [μm, ±SD]			
		Day 0	Day 15	Day 30	Day 60
HYDROGEL 1	temp. 4 °C	427.893 ± 4.340	424.623 ± 3.947	421.353 ± 5.096	418.083 ± 5.607
	temp.25 °C	423.567 ± 4.198	420.040 ± 4.016	416.513 ± 4.954	412.986 ± 5.676
	temp. 40 °C	428.780 ± 4.302	422.252 ± 3.858	415.724 ± 5.058	409.196 ± 5.518
HYDROGEL 2	temp. 4 °C	416.926 ± 4.271	416.628 ± 3.844	416.330 ± 5.027	416.032 ± 5.504
	temp. 25 °C	418.367 ± 4.138	418.015 ± 3.935	414.890 ± 4.894	411.765 ± 5.595
	temp. 40 °C	420.849 ± 4.328	415.566 ± 3.966	410.283 ± 5.084	405.000 ± 5.626
HYDROGEL 3	temp. 4 °C	423.492 ± 4.302	419.909 ± 3.820	416.326 ± 5.058	412.743 ± 5.480
	temp. 25 °C	421.478 ± 4.138	418.351 ± 3.931	415.224 ± 4.894	412.097 ± 5.591
	temp. 40 °C	423.947 ± 4.239	418.575 ± 3.959	413.203 ± 4.995	407.831 ± 5.619
HYDROGEL 4	temp. 4 °C	428.009 ± 5.340	424.739 ± 5.947	421.469 ± 4.696	418.199 ± 6.607
	temp. 25 °C	423.683 ± 5.198	420.156 ± 6.016	416.629 ± 4.554	413.102 ± 6.676
	temp. 40 °C	428.896 ± 5.302	422.368 ± 5.858	415.840 ± 4.658	409.312 ± 6.518
HYDROGEL 5	temp. 4 °C	417.042 ± 5.271	416.744 ± 5.844	416.446 ± 4.627	416.148 ± 6.504
	temp. 25 °C	418.483 ± 5.138	418.131 ± 5.935	415.006 ± 4.494	411.881 ± 6.595
	temp. 40 °C	420.965 ± 5.328	415.682 ± 5.966	410.399 ± 4.684	405.116 ± 6.626
HYDROGEL 6	temp. 4 °C	423.608 ± 5.302	420.025 ± 5.820	416.442 ± 4.658	412.859 ± 6.480
	temp. 25 °C	421.594 ± 5.138	418.467 ± 5.931	415.340 ± 4.494	412.213 ± 6.591
	temp. 40 °C	424.063 ± 5.239	418.691 ± 5.959	413.319 ± 4.595	407.947 ± 6.619

**Table 5:** Quantitative analysis of iridoid glycosides in the hydrogel formulations (2, 3, 5, and 6) after storage period of 60 days under various temperature conditions (4, 25, 40 °C).

	Temp. 4 °C		Temp. 25 °C		Temp. 40 °C	
	Concentration [μg/mL, ±SD]	% *	Concentration [μg/mL, ±SD]	% *	Concentration [μg/mL, ±SD]	% *
HYDROGEL 2	49.124 ± 0.131	1.75	48.971 ± 0.301	2.06	48.273 ± 0.423	3.45
HYDROGEL 5	4.946 ± 0.376	1.09	4.933 ± 0.213	1.34	4.857 ± 0.156	2.86
HYDROGEL 3	49.300 ± 0.225	1.40	48.998 ± 0.246	2.00	48.178 ± 0.238	3.64
HYDROGEL 6	4.451 ± 0.317	1.03	4.440 ± 0.267	1.12	4.371 ± 0.254	2.78

* average loss of active substance [%].

## Data Availability

The data presented in this study are available by authors upon request.
